# The impacts of maternal childhood adversity, stress, and mental health on child development at 6 months in Taiwan: A follow-up study

**DOI:** 10.1017/S0954579420000267

**Published:** 2021-08

**Authors:** Yi-Ting Chang, Jui-Ying Feng, Hsin-Yi Chang, Yu-Chun Chang, Chia-Kuei Lee

**Affiliations:** 1 Department of Nursing, College of Medicine, National Cheng Kung University, Taiwan; 2 Department of Nursing, National Cheng Kung University Hospital, College of Medicine, National Cheng Kung University, Taiwan

**Keywords:** adverse childhood experiences, intergenerational effect, perinatal depression, prenatal stress, psychosocial risk

## Abstract

Exposure to adverse childhood experiences (ACEs) is not only associated with one's adverse health outcomes in adulthood but also increases the risk of child developmental problems in offspring. However, the mechanisms involved in the transmission of the effects of maternal ACEs to the offspring largely remain unexplored. This study sought to identify possible psychosocial pathways of intergenerational effects of maternal ACEs on child development at 6 months. Data from a longitudinal study on maternal childhood adversity and maternal psychosocial risk during pregnancy as well as maternal mental health problems and child development at 6 months postnatal were used. Structural equation modeling with bootstrapping was used to estimate the indirect effects of maternal ACEs on child development at 6 months. The model showed that maternal ACEs indirectly influenced offspring's development via maternal stressful events during pregnancy and pre- and postnatal mental health problems. This finding highlights the possible interventions at the prenatal and postnatal periods. Early identification of women who have ACEs or who are at psychosocial risk during pre- and postnatal periods is critical to provide interventions to buffer those negative effects on offspring's development. Future studies are needed to longitudinally assess the effects of maternal ACEs on child development over time.

Adverse childhood experiences (ACEs), such as child maltreatment and family dysfunction, are stressful or traumatic events, which increase the risk of health, social, and behavioral problems throughout a person's life span (Austin, 2018; Centers for Disease Control and Prevention, 2016). Findings from a systematic review and meta-analysis study revealed that exposure to ACEs is associated with adult adverse outcomes, such as mental illness, substance use, sexual risk taking, obesity, physical inactivity, interpersonal and self-directed violence, and chronic diseases and conditions (Hughes et al., 2017). Moreover, in a review by Bowers and Yehuda (2016), maternal ACEs increased the risk of the offspring manifesting negative physical, behavioral, and cognitive outcomes. The effect of maternal ACEs on the offspring's health outcomes could be due to intergenerational transmission via maternal pre- and postnatal physical and mental health (Kumar et al., 2018; Letourneau et al., 2019; Madigan, Wade, Plamondon, Maguire, & Jenkins, 2017). However, most studies exploring the pathway linking maternal ACE and offspring's health outcomes were only focused on postpartum maternal health (Folger et al., 2018; Rieder et al., 2019; Sun et al., 2017) or investigated prenatal and postnatal maternal health separately (Letourneau et al., 2019). Therefore, it is unclear whether the effects of maternal health persist from the prenatal to the postnatal period. Given the significant negative effects of maternal ACEs on offspring's health outcomes, identifying the mechanisms of intergenerational effects of ACEs on offspring's development is essential for a more comprehensive view of child health and development.

Researchers have shown both direct (Folger et al., 2018; Sun et al., 2017) and indirect effects of maternal ACEs on child development (Buss et al., 2017; Letourneau et al., 2019; Thomas, Letourneau, Campbell, & Giesbrecht, 2018). For the indirect effects, two mechanisms involved in transmitting the effects of maternal ACEs to the offspring have been proposed: biological (e.g., endocrine and immune systems, and hypothalamic–pituitary–adrenal [HPA] axis function) and psychosocial (e.g., depression, stress, affective symptoms, and social support) mechanisms. For example, one important biological mechanism underlying the relationship between maternal ACEs and offspring's health is the HPA axis function. Researchers have demonstrated that maternal HPA axis function during pregnancy mediated the effects of maternal ACEs on infant HPA axis reactivity (Beijers, Buitelaar, & de Weerth, 2014; Thomas et al., 2018). Activation of infants’ HPA axis affects their cognitive and social-emotional development and potentially changes their telomere length, which is associated with age-related disease and mortality (Laurent, 2017; Nelson, Allen, & Laurent, 2018).

In contrast, other researchers suggested the psychosocial mechanism as underlying the transmission of the effects of maternal ACEs to the offspring (Collishaw, Dunn, O'Connor, Golding, & Avon Longitudinal Study of Parents and Children Study Team, 2007; Miranda, de la Osa, Granero, & Ezpeleta, 2013). Many studies have suggested that maternal pre- and postnatal psychosocial risk (e.g., stress during pregnancy, anxiety, and depression) are the potential psychosocial mechanisms underlying the impacts of ACEs on offspring's development (Racine, Madigan, Plamondon, Hetherington, et al., 2018; Racine, Plamondon, Madigan, McDonald, & Tough, 2018). Pregnant women with childhood adversity have a higher tendency to develop pre- and postnatal stress and mental health problems compared to women without a history of ACEs (Racine, Madigan, Plamondon, McDonald, et al., 2018; Seng et al., 2013). Moreover, both single and combined effects of maternal ACEs, stress, and mental health problems jeopardize offspring socioemotional and cognitive development (Madigan et al., 2018; Racine, Plamondon, Nadigan, McDonald, & Tough, 2018; Tarabulsy et al., 2014).

Although the association between maternal ACEs and child development was investigated, those studies were usually conducted among children aged older than 12 months or only focused on the temperament and socioemotional functioning of children aged 6 months (Enlow et al., 2017; McDonnell & Valentino, 2016). Considering that child development is a continuous process, early development is an important foundation of future learning and physical and mental health. The emerging abilities of self-sitting, object exploration, reaching behavior, and grasping action by 6 months are the first significant milestones of early child development, and are related to children's cognitive, motor, and social development (Daum, Prinz, & Aschersleben, 2011; Hopkins & Rönnqvist, 2002; Soska & Adolph, 2014). Researchers have found that infants at 6 months with motor delay had high risk of autism spectrum disorders (Libertus, Sheperd, Ross, & Landa, 2014) and potential for future social, language, and communication delays (Bhat, Galloway, & Landa, 2012; Flanagan, Landa, Bhat, & Bauman, 2012). Evaluating the child's developmental milestones at 6 months is particularly important to identify the potential developmental delays and minimize later developmental problems. Early identification and intervention can help improve health outcomes for children with developmental delay (Spittle & Treyvaud, 2016).

There is a need to better understand the potential pathways linking maternal childhood adversity to child development through both prenatal and postnatal psychosocial risk. In addition, the effect of maternal ACEs and mental health problems on other aspects of child development at 6 months, such as children's gross motor, fine motor, language, and social development, needs to be explored. Thus, the present study aimed to identify possible psychosocial mechanisms of the transmission of maternal ACEs to infants at 6 months of age (see Figure 1 for the proposed model). We hypothesized that maternal ACEs had negative impacts on prenatal and postnatal psychosocial risk, and their offspring's development. More specifically, we determined whether the effects of maternal childhood adversity on their children's development (i.e., gross motor, fine motor, language, and social development) at 6 months were mediated through prenatal maternal stress and mental health problems (i.e., depression and anxiety) and postnatal mental health problems. These findings would provide a starting point for developing an intervention for pregnant women and their families to support and benefit traumatized mothers and their children.

**Figure 1.**
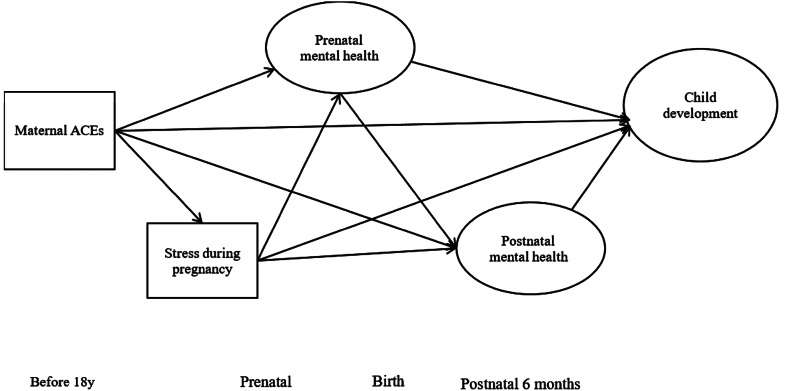
Proposed model.

Although maternal stress during pregnancy and prenatal mental health may have a potential bidirectional relationship, we considered that maternal stress (stressful events during pregnancy) was more likely to affect prenatal mental health rather than vice versa in our study based on the literature. A systematic review conducted by Lancaster et al. (2010) revealed that life stresses (or negative life events) may be potential predictors of an increased risk of depressive symptoms during pregnancy. Mitchell and Ronzio (2011) also showed that stressful events increased the risk for maternal depression and symptoms of anxiety in African American mothers. Similarly, Coussons-Read (2013) mentioned that exposure to stress (or negative life event) may be the etiology of depression. Therefore, the influence of prenatal stress on infant health and development may be due to the increased maternal risk of anxiety and depression during pregnancy.

## Method

### Study design

This study was part of a Longitudinal Study of Mother and Children's Health (LSMACH), which aimed to explore the effect of maternal childhood adversity on mothers’ and children's health outcomes in Taiwan. Pregnant women seeking prenatal care in their second or third trimester were recruited from the prenatal clinic in a medical center in Southern Taiwan, where 1,200–1,400 deliveries take place yearly. In the LSMACH, data were collected at four time points including baseline (between 13 and 37 weeks of gestation), and when infants were aged 1, 6, and 18 months. The pregnant women were enrolled between January 2016 and September 2016; follow-up continued until December 2018. A self-report questionnaire containing demographic information and questions regarding maternal childhood adversity and maternal psychosocial risk (stress, depression, and anxiety) was administered at baseline. At 1, 6, and 18 months postnatal follow-ups, questionnaires about maternal mental health problems (depression and anxiety) and child development were administered. An approval of all study activities was obtained from the university institutional review board (No. A-ER-104-253). In the present study, data on maternal childhood adversity and maternal psychosocial risk at baseline as well as maternal mental health problems and child development at 6 months postnatal were used.

### Participants

In the LSMACH, the eligible participants were pregnant women in their second or third trimester, 18 years and above, and able to complete the Chinese questionnaire. A total of 295 pregnant women were recruited from the prenatal clinic in a medical center in Southern Taiwan. Of these, 210 women had completed at least one valid postnatal follow-up (postnatal 1 month: *n* = 142, 6 months: *n* = 130, and 18 months: *n* = 127; see Appendix A in the online-only Supplementary materials for the flow chart of participants). Participants included in the present study were 130 mother–child dyads who completed the questionnaires at both baseline and 6 months postnatal. The retention rate at the 6-month follow-up assessment was 44%. Reasons for loss to follow-up included abortion and child death (*n* = 7, 4.2%), withdrawal (*n* = 10, 6.1%), and participants being unreachable because their phone was off (*n* = 59, 35.8%) or out of service (*n* = 11, 6.7%). Nearly half of the participants (*n* = 78, 47.3%) who were contacted agreed to participate in the 6-month follow-up assessment but did not return the questionnaires. There were no demographic differences (i.e., educational level, marital status, employment status, household monthly income, history of pregnancy, pregnancy intention, or complications during pregnancy) between those women who were lost to follow-up and those in this analysis. However, the women who were lost to follow-up (32.8 ± 4.0 years) were slightly younger than those in this analysis (33.7 ± 4.2 years), *t* (293) = 2.05, *p* = .041.

### Measurement

#### Maternal childhood adversity

The 14-item revised ACE scale (Finkelhor, Shattuck, Turner, & Hamby, 2015) was used to measure pregnant women's experiences of psychological abuse, physical abuse, sexual assault, emotional neglect, physical neglect, witnessing mother being treated violently, household substance abuse, household mental illness, parental separation or divorce, having an incarcerated household member, being in a family of low socioeconomic status, peer victimization, peer isolation/rejection, and exposure to community violence before age 18. Each item was responded to dichotomously, *Yes* (experienced the event) and *No* (did not experience the event). The number of “Yes” answers was summed to obtain a total score. A higher total score indicates women had a greater number of ACEs. The Kuder–Richardson 20 coefficient was .76 in the present sample.

#### Stressful events during pregnancy

Ten items were adopted from the Social Readjustment Rating Scale (SRRS; Holmes & Rahe, 1967) and the Taiwan Intimate Partner Violence Danger Assessment (Wang, 2012) to measure the stressful life or traumatic events during pregnancy. Items selected from the SRRS were related to major stressful life events, including marital divorce or separation; death of partner, family members, or friends; arguing with partner or partner's family members; sickness or serious illness in the family; and losing or changing a job. Those items indicate relatively high-stress events on the SRRS and are related to the context of pregnancy. Items selected from the Taiwan Intimate Partner Violence Danger Assessment form were related to physical, psychological, and sexual abuse by partner, including violence against children by partner, actual or threatened death or serious injury by partner, and forced sexual activity by partner. Each item was coded as 1 if the woman had experienced the event and as 0 if she had never experienced the event. Higher total scores indicate more stressful events during pregnancy.

#### Depression

The 10-item Chinese version of the Edinburgh Postnatal Depression Scale (EPDS) was used to measure women's prenatal and postnatal depressive symptoms. The EPDS has been widely used and validated in women's postpartum as well as prenatal and perinatal periods. Participants were asked to check off one of four possible answers that best described their mood during the past week. Responses are scored 0, 1, 2, or 3 according to increased severity of the symptom. For example, the scores for “I felt sad or miserable” were 3 = *most of the time*, 2 = *quite often*, 1 = *not very often*, and 0 = *not at all*. The total score ranges from 0 to 30, with a higher score indicating possible depression. Cronbach's α was .87 for the Chinese version of the EPDS, and concurrent validity was .79 with the Beck Depression Inventory (Heh, 2001). Cronbach's α was .81 in this study.

#### Anxiety

The Chinese version of the Generalized Anxiety Disorder 7-item Scale was used to measure how frequently women felt anxious over the past 2 weeks. Responses were scored ranging from 0 = *not at all*, 1 = *a few days*, 2 = *more than a week*, to 3 = *nearly every day*. The total score ranges from 0 to 21, with a higher score indicating more severe generalized anxiety disorder symptoms. Cronbach's α was .89 for the Chinese version of the Generalized Anxiety Disorder 7-item Scale (Tong, An, McGonigal, Park, & Zhou, 2016). Cronbach's α was .89 in this study.

#### Child development

The parent-report Taiwan Birth Cohort Study–Developmental Instrument (TBCS-DI) includes four dimensions of development: gross motor, fine motor, language, and social development at 6, 18, 36, and 60 months. We used the 6-month scale (26 items) in this study. A 3-point Likert scale (*always*, *sometimes*, and *never*) was used, with higher scores implying better development. The potential scores were 9–27, 7–21, 7–21, and 3–9 for gross motor, fine motor, language, and social development, respectively. TBCS-DI is an efficient developmental screening instrument. Good reliability of the TBCS-DI and its good concurrent validity with the Bayley Scale of Infant Development–II has been demonstrated in the literature (Lung, Chiang, Lin, Lee, & Shu, 2010; Lung, Shu, Chiang, & Lin, 2008). In this study, Cronbach's α for the four subscales were .75, .60, .74, and .60 for gross motor, fine motor, language, and social development, respectively.

#### Demographics of mothers and offspring

Demographic characteristics of mothers included maternal age (in years), educational level (below high school, high school, or college and above), marital status (married/not married), employment status (yes/no), household monthly income (NTD <28,000 [low], 28,000─70,000 [medium], and ≥70,000 [high]), history of pregnancy, pregnancy intention (planned pregnancy/unplanned pregnancy), and complications during pregnancy (had prenatal complications/none). Demographic characteristics of offspring were gender (male/female), gestational age (in weeks), and body weight at birth (in grams).

### Data analysis

Descriptive statistics were used to describe demographics, maternal childhood adversity, stressful events during pregnancy, pre- and postnatal depression and anxiety, and child development at 6 months. The total scores for each variable were calculated by multiplying the total number of items and the mean scores of valid items. Pearson's correlation and point-biserial correlation coefficients were used to examine the associations among demographics, maternal childhood adversity, stressful events during pregnancy, pre- and postnatal depression and anxiety, and child development at 6 months. Structural equation modeling was performed using Mplus 8.0 (Muthén and Muthén, Los Angeles, CA, USA) to examine the direct and indirect effects of maternal childhood adversity on child development at 6 months. Maternal pre- and postnatal depression and anxiety were used as indicators for the latent constructs of pre- and postnatal maternal mental health separately. Four dimensions of child development (i.e., gross motor, fine motor, language, and social development) were used as indicators for the latent construct of child development (Lung et al., 2008). The measurement model was tested by a confirmatory factor analysis to verify whether the indicators loaded significantly (*p* < .001) onto the expected latent constructs. Maternal parity, premature birth, and offspring's sex were used as covariates for prenatal maternal mental health, postnatal maternal mental health, and child development, respectively.

The model parameters were estimated using the maximum likelihood estimate. Model fit was assessed using chi-square ratio (χ^2^/*df*), the root mean square error of approximation (RMSEA), the standardized root mean square residual (SRMR), and the comparative fit index (CFI). Values of .08 or lower for the RMSEA (Fabrigar, MacCallum, Wegener, & Strahan, 1999) and SRMR (Hu & Bentler, 1999), of .95 or above for the CFI (Hu & Bentler, 1999), and between 1 and 3 for the χ^2^/*df* (Hair, Anderson, Tatham, & Black, 1998) suggest good model fit. Missing data were handled with full information maximum likelihood. In addition, the indirect effects of maternal childhood adversity on child development through maternal prenatal stress and mental health were estimated using bias-corrected bootstrapping with 2,000 bootstrap samples. The significant indirect effects were considered when the 95% bias-corrected confidence interval (CI) did not include 0.

## Results

### Demographics

The data of 130 mother–child dyads were analyzed in this study. Table 1 shows the demographics of mothers and offspring. The mean age of women was 33.7 years (*SD* = 4.2). Most women were married (96.9%) and employed (77.5%), and had a college degree or higher level of education (95.3%). Most women had a high monthly household income of more than NTD 70,000 (47.3%), which indicated that household income was more than expenditure and that they were considered high-income earners. Most pregnancies were planned (68.5%) and without prenatal complication (90%). Moreover, the number of children for both sexes is equal. Fourteen children (10.8%) were born preterm and 17 (13.1%) were of low birth weight, with 8 being both premature and low birth weight infants.

**Table 1.** Demographic information (n = 130)

Demographic	*n* (%)
**Mother's characteristics**	
Age (year)	33.7 ± 4.17
Education	
High school or lower	6 (4.7)
Bachelor	88 (68.2)
Master/doctor	35 (27.1)
Household monthly income	
≤27,999 NTD (low)	12 (9.3)
28,000–69,999 NTD (medium)	56 (43.4)
≥70,000 NTD (high)	61 (47.3)
Employment status	
Employed	100 (77.5)
Unemployed	29 (22.3)
Artificial insemination	9 (6.9)
Primigravida	77 (59.2)
Miscarriage	19 (14.6)
Artificial abortion	18 (13.8)
Unplanned pregnancy	41 (31.5)
Prenatal complications	13 (10.0)
**Child's characteristics**	
Gender	
Female	65 (51.6)
Male	61 (48.4)
Gestational age (weeks)	38.3 ± 1.66
Premature birth	14 (10.8)
Birth weight (g)	2925.0 ± 462.73
Low birth weight	17 (13.1)

### Maternal ACEs, maternal mental health, and offspring's outcomes

More than a quarted of mothers had ACEs and over a third experienced stressful events during pregnancy (Table 2). The most common stressful event during pregnancy was conflicts with family members (*n* = 22, 16.9%), which usually happened with one's partner (*n* = 10) or partner's family members (*n* = 8).

**Table 2.** Maternal adversity childhood experiences and prenatal stress (n = 130)

Variables	*n* (%)
**Adversity childhood experiences**	35 (26.9)
Emotional abuse	9 (6.9)
Physical abuse	5 (3.8)
Sexual assault	5 (3.8)
Emotional neglect	7 (5.4)
Physical neglect	1 (0.8)
Parental divorce/separation	13 (10)
Mother treated violently	6 (4.6)
Family drug/alcohol problem	2 (1.5)
Family mental illness	10 (7.8)
Parent ever go to prison	1 (0.8)
Peer victimization	3 (2.3)
Peer social isolation	6 (4.6)
Exposure to community violence	2 (1.5)
Low socioeconomic status	5 (3.8)
**Stressful events during pregnancy**	49 (37.7)
Marital divorce or separation	2 (1.5)
Death of family members or friends	21 (16.2)
Conflicting with family members	22 (16.9)
Had sickness or serious illness	1 (0.8)
Sickness or serious illness in the family	6 (4.6)
Losing or changing a job	8 (6.2)
Violence against children by partner	0 (0.0)
Injured by partner	1 (0.8)
Threatened death by partner	1 (0.8)
Forced sex behaviors by partner	0 (0.0)

Table 3 indicates the relationships among demographics of mothers and offspring, maternal ACEs, stressful events during pregnancy, depression and anxiety, and their offspring's development. Primigravidas had lower prenatal depression than multiparas (*r* = –.19, *p* = .028). Mothers who delivered prematurely had high postnatal depression (*r* = .21, *p* = .016) and prenatal anxiety (*r* = .24, *p* = .007). Maternal ACEs were positively correlated with stressful events during pregnancy (*r* = .27, *p* = .002) and prenatal depression (*r* = .25, *p* = .004). The correlations of maternal ACEs with prenatal anxiety, postnatal depression and anxiety, and child development were insignificant. In addition, stressful events during pregnancy positively correlated with pre- and postnatal depression and anxiety (*r =* .24 ~ .35, *p* ≤ .001 ~ *p* = *.005*) and negatively correlated with offspring's fine motor, language, and social development (*r* = –.21 ~ –.17, *p* = .016 ~ .043). Pre- and postnatal maternal depression and anxiety were negatively correlated with offspring's gross motor, fine motor, and language development (*r* = –.40 ~ –.23, *p* < .001 ~ *p* =.006). However, postnatal depression and pre- and postnatal anxiety (*r* = –.27 ~ –.19, *p* = .002 ~ .025) were negatively correlated with offspring's social development, but prenatal depression was not (*p* = .106).

**Table 3.** Correlations among maternal ACEs, maternal mental health, and child development (n = 130)

Variables	*M* ± *SD*	Range	1	2	3	4	5	6	7	8	9	10	11	12	13	14
1. Maternal age			1													
2. Primigravida			–.271**	1												
3. Child sex			.065	.123	1											
4. Premature			.019	.089	–.008	1										
5. Low birth weight			.035	.142	.019	.452**	1									
6. Maternal ACEs	0.6 ± 1.37	0–9	–.041	–.051	–.007	.070	–.067	1								
7. Stressful events during pregnancy	0.5 ± 0.70	0–3	.019	–.091	–.097	–.104	–.014	.269**	1							
8. Prenatal depression	7.6 ± 3.87	0–20	.034	–.193*	.024	.088	.052	.249**	.292**	1						
9. Postnatal depression	7.7 ± 4.35	0–26	.037	–.081	–.072	.214*	.163	.166	.346**	.627**	1					
10. Prenatal anxiety	2.7 ± 2.93	0–14	.047	–.102	.121	.238**	.304**	.059	.243**	.683**	.480**	1				
11. Postnatal anxiety	3.1 ± 3.27	0–19	–.013	–.069	.016	.162	.181*	.127	.329**	.541**	.792**	.574**	1			
12. Gross motor	25.6 ± 2.66	11–27	–.109	.065	–.105	–.126	–.163	–.023	–.161	–.247**	–.362**	–.339**	–.319**	1		
13. Fine motor	19.5 ± 2.03	8–21	–.010	–.032	–.173	–.173	–.216*	–.126	–.207*	–.241**	–.402**	–.306**	–.392**	.644**	1	
14. Language	19.2 ± 2.25	8–21	.049	.017	–.139	–.060	–.205*	.070	–.174*	–.235**	–.342**	–.295**	–.351**	.526**	.646**	1
15. Social development	8.3 ± 1.21	3–9	–.130	–.013	.016	–.022	–.103	.021	–.209*	–.139	–.263**	–.193*	–.269**	.511**	.636**	.538**

*Note*: ACEs, adverse childhood experiences. *p < .05. **p < .01.

### The effect of maternal ACEs on their offspring's development

**Figure 2.**
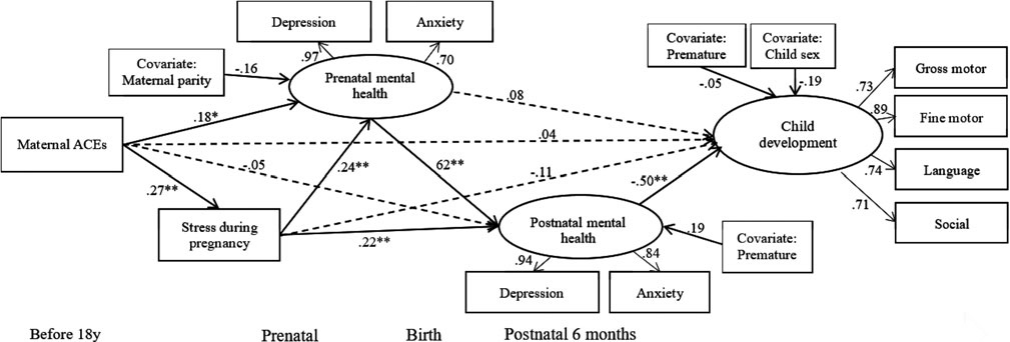
Pathways of maternal adverse childhood experiences (ACEs) on child development at 6 months through maternal psychosocial risk (stress, depression, and anxiety). Standardized coefficients were presented. Solid lines indicate significant paths; dashed lines indicate nonsignificant paths. *p < .05. **p < .01.

The result of structural equation modeling with standardized coefficients is presented in Figure 2. The model explained 29.4% of the variance and showed acceptable fit based on multiple fit statistics: χ^2^/*df* = 1.53, CFI = .955, RMSEA = .064, and SRMR = .057. Maternal ACEs were associated with maternal stressful events during pregnancy (β = .27, *SE* = .08, *p* = .001) and prenatal mental health (β = .18, *SE* = .09, *p* = .045). Maternal stressful events during pregnancy was associated with prenatal mental health (β = .24, *SE* = .09, *p* = .045) and postnatal mental health (β = .22, *SE* = .08, *p* = .004). Maternal prenatal mental health was associated with postnatal mental health (β = .62, *SE* = .09, *p* < .001), which was associated with child development at 6 months (β = –.50, *SE* = .13, *p* < .001). In terms of indirect effects, results indicated three significant pathways linking maternal ACEs to child development at 6 months: (a) through maternal stressful events during pregnancy and maternal postnatal mental health (β = –.03, *SE* = .02, 95% CI [–.116 ~ –.004]); (b) through maternal pre- and postnatal mental health (β = –.05, *SE* = .04, 95% CI [–.209 ~ –.011]; and (c) through maternal stressful events during pregnancy, and maternal pre- and postnatal mental health (β = –.02, *SE* = .02, 95% CI [–.114 ~ –.002]).

## Discussion

We investigated possible psychosocial pathways in explaining the transmission of maternal experiences of childhood adversity to offspring, and its negative effect on offspring's development at 6 months. The findings of structural equation modeling in this study partly supported our hypothesis that maternal ACEs were directly associated with higher levels of stress during pregnancy and prenatal mental health (i.e., depression and anxiety) and indirectly associated with higher levels of postnatal mental health and child development at 6 months. Mothers who experienced more adversities in childhood had higher psychosocial risk (i.e., stress, depression, and anxiety) during both pregnancy and the postnatal period, which, in turn, led to their offspring's poor developmental outcomes at 6 months of age. The results supported the psychosocial pathway (pre- and postnatal maternal mental health), explaining the association between maternal ACEs and developmental risk in the next generation.

Maternal ACEs and prenatal psychosocial risk had indirect effects on child development via postnatal mental health. This finding is consistent with previous literature indicating that adverse events in life, high perceived stress, and mental health problems during pregnancy were high-risk factors for postpartum mental health problems and adverse offspring development (O'Connor, Monk, & Fitelson, 2014; Seng et al., 2013). Researchers have shown that women with postnatal depression were less likely to engage in parenting or caregiving practices, which increases the risk of developmental delays (such as in gross motor functioning and problem solving) in children aged 6–18 months (Vameghi, Amir Ali Akbari, Sajjadi, Sajedi, & Alavimajd, 2015). Therefore, maternal prenatal stress and mental health play a critical role in child development. The continuity or escalation of maternal mental health problems from the prenatal to the postnatal period would contribute to adverse child development.

Although the independent effects of maternal ACEs and mental health on offspring's development were found in previous studies, how maternal ACEs function with prenatal and postnatal mental health in influencing offspring's development still remains to be defined (Folger et al., 2018; Racine, Plamondon, et al., 2018). Racine, Plamondon, et al. (2018) conducted a cohort study with 1,994 mother–infant dyads and found an indirect effect of maternal ACEs on offspring's development at 1 year of age via prenatal psychosocial risk, but not postnatal psychosocial risk at 4 months postpartum. In another cohort study conducted by Folger et al. (2018), among 311 mother–child and 122 father–child dyads, only maternal mental health at the postnatal period was measured. Their findings revealed that maternal ACEs had direct effects on maternal postnatal depressive symptoms at 2 months postpartum and offspring's development at 2 years of age. However, maternal postnatal depressive symptoms did not mediate the effects of maternal ACEs on offspring's development. In the present study, the pathway from maternal ACEs to offspring's development was via prenatal stress and mental health problems first, and then via postnatal mental health problems. Therefore, further studies that estimate maternal mental health at both the pre- and postnatal periods are needed to clarify these pathways via maternal mental health in transmitting maternal ACEs to infants.

While the findings did support the indirect effects of ACEs on child development at 6 months, the effect size was small (*R*^2^ = .29). This might be due to the limited measure of child development in the LSMACH study. Although the measure of child development targeted the significant developmental milestones, this measure only covered the developmental aspects of gross motor, fine motor, language, and social development. Nonetheless, the impacts of maternal ACEs and prenatal mental health problems on the offspring's cognitive and socioemotional development are of the most concern, especially if through the psychosocial mechanism (Kingston, McDonald, Austin, & Tough, 2015; Treat, Sheffield-Morris, Williamson, & Hays-Grudo, 2019). Moreover, some unmeasured factors in this study, including parenting roles, parenting behaviors, and parent–child interactions, may also be important influence factors of child development (Chiang, Lin, Lee, & Lee, 2015; Glascoe & Leew, 2010). Future studies are needed to incorporate these aspects of child development and include other potential influence factors to have a more integrated understanding of the impacts of maternal ACEs on child development.

Our findings also showed that maternal prenatal stress was strongly associated with prenatal depression and anxiety. The most common stressful life or traumatic event during pregnancy that women experienced was conflict with partner or partner's family members. Researchers have found that dissatisfaction with the partner, partner conflict, low partner support, and low social support increased maternal emotional distress (Cheng et al., 2016; Røsand, Slinning, Eberhard-Gran, Røysamb, & Tambs, 2011; Tyrlik, Konecny, & Kukla, 2013). In Chinese culture, in-law family conflict also plays an influential role in intimate partner violence against women (Choi, Chan, & Brownridge, 2010). Intimate partner violence during pregnancy affected woman's physical and mental health and increased the risk of low birth weight infants and neonatal death (Chen & Lin, 2015). Furthermore, researchers have shown that resilience is a protective factor for pregnant women to overcome adversities and cope with stress, depression, and anxiety during pregnancy (Lévesque & Chamberland, 2016; Ma et al., 2019). Taken together, interventions that target family and social support and/or enhance pregnant women's resilience may be promising strategies to mitigate maternal psychosocial risk (stress, depression, and anxiety) and improve women's pregnancy outcomes.

In addition, the findings indicate that maternal mental health mediated the effects of maternal ACEs and prenatal stress on child development. Researchers have found that women with ACEs had higher perinatal depressive symptoms than those without ACEs (Mersky & Janczewski, 2018). Women with a history of childhood adversity were more likely to report depressive symptoms during both the antenatal and postnatal periods (Ångerud, Annerback, Tyden, Boddeti, & Kristiansson, 2018; Mersky & Janczewski, 2018). Therefore, the identification of pregnant women with anxiety and depression will be essential to the provision of timely support for those women and their family members. Moreover, addressing the issues regarding the factors associated with perinatal mental health problems during the antenatal and postnatal periods (e.g., prenatal clinic, well-baby clinic, and antenatal classes) will be important in promoting the well-being of mothers and their babies. Educating pregnant women about their maternal role, expected changes in their physical appearance, effects of the changes in hormone levels, the potential influence of past and present pregnancy complications to their current pregnancy, the importance of partner support and social support, as well as asking them about their attitudes and beliefs toward motherhood, is vital in the reduction and prevention of perinatal mental health problems (Biaggi, Conroy, Pawlby, & Pariante, 2016). Furthermore, a family-centered approach to engage fathers or partners and other family members in taking care of the mothers and their babies would be beneficial in enhancing family relationships by mobilizing support and resources from the partner and the entire family as a whole (Rahman et al., 2013; Samal, 2016). This, in turn, may actually improve the outcome of the children. Bergman, Sarkar, Glover, and O'Connor (2010) have also found that if infants experience postnatal environments enriched with sensitive and response caregiving behavior as well as secure attachment, the negative influence of maternal prenatal stress on child outcomes may be buffered.

In addition, the key role of healthcare providers in promoting maternal mental health and the child's well-being should be highlighted. Aside from identifying high-risk pregnant women, preventing perinatal mental health problems is also important. Researchers have found that the use of active psychotherapeutic techniques or psychoeducational approaches in conjunction with conventional prenatal care may help pregnant women recognize and manage depression symptoms, enhance problem-solving skills, adapt positive ways of thinking, promote social support and a healthy lifestyle, and implement useful behavior strategies—all of which may be effective in reducing perceived stress and anxiety, which, in effect, may improve maternal mental health (Atif, Lovell, & Rahman, 2015; Barlow et al., 2010; Beddoe & Lee, 2008). Thus, professional trainings need to continuously improve healthcare providers’ knowledge and abilities in recognizing high-risk pregnant women because the early intervention for perinatal mental health problems is a main priority (Legere et al., 2017).

A few limitations should be noted in this study. The self-reported data of mothers may be biased by social desirability. The childhood adversity experiences and stressful events during pregnancy may be underreported. Moreover, the use of questionnaires to measure child development based on mothers’ report would bias the data. However, the child developmental instrument used in this study is a culturally sensitive one and includes comprehensible items for Taiwanese parents. Furthermore, the small sample size in this study lacks sufficient statistical power to detect the significance of effect sizes. Future studies with a large sample size and objective measures of child development are needed to clarify these findings. In addition, because only two time points were examined, the causal direction of the effects cannot be fully established. Because the majority of the women in this study were from one medical center, well educated, and had high household family incomes, the generalizability of these findings is limited. While the lower response rate (<50%) at the 6-month follow-up may potentially raise the selection bias, it is less likely to be the case in this study. This is because our examination has shown, excepting women who were lost to follow-up were slightly younger, no other difference in baseline data and demographics between women who were lost to follow-up and those who were analyzed in this study.

In sum, the findings of this study offer preliminary support for the mechanism involved in the transmission of maternal ACEs to the offspring through maternal psychosocial risk. These findings have some practical and research implications. Primary care providers in prenatal care clinics and well-child visits are in the best position to assess maternal childhood adversity and mental health problems. Early identification of women who had childhood adversities and/or mental health problems during prenatal and postnatal visits can ensure that emotional support and relevant interventions are provided to promote their quality of childcare. Future research with large and diverse populations from different levels of socioeconomic status to clarify the effects of maternal ACEs on child development is needed. In addition, a longitudinal study with a long follow-up period would be helpful to explore the long-term impacts on child health and developmental outcomes over time.

### Conclusions

Maternal psychosocial risk during pregnancy and the postnatal period serves as a mechanism in transmitting the effects of maternal ACEs on offspring at 6 months. The findings of this study highlight the importance of interventions at different stages of this psychosocial pathway. Early identification of women with ACEs and assessment of pre- and postnatal maternal mental health problems are critical strategies to identify the children at risk. This would also provide the opportunity to initiate early interventions to promote maternal mental health and prevent/mitigate the adverse child developmental outcomes.

## Supplementary material

For supplementary material accompanying this paper visit https://doi.org/10.1017/S0954579420000267. click here to view supplementary material
