# Supplementary material for: The impacts of maternal childhood adversity, stress, and mental health on child development at 6 months in Taiwan: A follow-up study
Source: Dev Psychopathol. 2021 Aug;33(3):970–9. doi: 10.1017/S0954579420000267 (PMC8374618; doi:10.1017/S0954579420000267)
Supplement: Supplementary file 1 [file S0954579420000267sup001.docx]

Appendix

Flow chart of participants

Recruited into the study from the prenatal clinic

*n* = 295

Completed the questionnaires at postnatal 1 month, *n* = 142

Reasons for loss to follow-up:

Abortion and child death (*n* = 5, 3.3%)

Withdrawal (*n* = 3, 2.0%)

Being unreachable because their phone was off (*n* = 77, 50.3%) or

out of service (*n* = 2, 1.3%)

Agreed to participate in the one-month follow-up assessment but did not

return the questionnaires (*n* = 66, 43.1%)

Completed the questionnaires at postnatal 6 months, *n* = 130

Reasons for loss to follow-up:

Abortion and child death (*n* = 7, 4.2%)

Withdrawal (*n* = 10, 6.1%)

Being unreachable because their phone was off (*n* = 59, 35.8%) or

out of service (*n* = 11, 6.7%)

Agreed to participate in the six-month follow-up assessment but did not

return the questionnaires (*n* = 78, 47.3%)

Completed the questionnaires at postnatal 18 months, *n* = 127

Reasons for loss to follow-up:

Abortion and child death (*n* = 7, 4.2%)

Withdrawal (*n* = 10, 6.0%)

Being unreachable because their phone was off (*n* = 40, 23.8%) or

out of service (*n* = 11, 6.5%)

Agreed to participate in the six-month follow-up assessment but did not

return the questionnaires (*n* = 100, 59.5%)
